# The 28 Ser Amino Acid of Cucumber Mosaic Virus Movement Protein Has a Role in Symptom Formation and Plasmodesmata Localization

**DOI:** 10.3390/v13020222

**Published:** 2021-01-31

**Authors:** Réka Sáray, Attila Fábián, László Palkovics, Katalin Salánki

**Affiliations:** 1 Centre for Agricultural Research, Plant Protection Institute, Herman Ottó Street 15., H-1022 Budapest, Hungary; saray.reka@atk.hu; 2 Department of Plant Pathology, Faculty of Horticultural Science, Szent István University, Villányi Street 29-43., H-1118 Budapest, Hungary; palkovics.laszlo@szie.hu; 3 Centre for Agricultural Research, Agricultural Institute, Brunszvik Street 2, H-2462 Martonvásár, Hungary; fabian.attila@atk.hu

**Keywords:** cucumber mosaic virus, movement protein, plasmodesmata localization signal, cell-to-cell movement, host plant-virus interaction

## Abstract

Cucumber mosaic virus (CMV, *Cucumovirus*, Bromoviridae) is an economically significant virus infecting important horticultural and field crops. Current knowledge regarding the specific functions of its movement protein (MP) is still incomplete. In the present study, potential post-translational modification sites of its MP were assayed with mutant viruses: MP/S28A, MP/S28D, MP/S120A and MP/S120D. Ser28 was identified as an important factor in viral pathogenicity on *Nicotiana tabacum* cv. Xanthi, *Cucumis sativus* and *Chenopodium murale*. The subcellular localization of GFP-tagged movement proteins was determined with confocal laser-scanning microscopy. The wild type movement protein fused to green fluorescent protein (GFP) (MP-eGFP) greatly colocalized with callose at plasmodesmata, while MP/S28A-eGFP and MP/S28D-eGFP were detected as punctate spots along the cell membrane without callose colocalization. These results underline the importance of phosphorylatable amino acids in symptom formation and provide data regarding the essential factors for plasmodesmata localization of CMV MP.

## 1. Introduction

Despite the great structural diversity of plant viral movement proteins (MPs), all of them have similar primary functions and they play a crucial role in the establishment of virus infection: They are (predominantly but not exclusively) responsible for the cell-to-cell movement of the viral genome and invading the non-infected neighboring cells. Several categories were defined based on the structural and functional characteristics of plant viral MPs. According to the number of encoded movement proteins, four groups can be differentiated [1]. Cucumber mosaic virus (CMV) is a member of the ‘30K superfamily’ group. All viruses in this category (except the bipartite begomoviruses) contain one movement protein and share a core domain in their structure containing a series of β-elements with an α-helix at both sides [2]. Some of them require the CP for effective cell-to-cell or long-distance movement [3,4] such as CMV. Some members of the 30K group form tubules through plasmodesmata (PD) while others transport the viral genome by increasing the size exclusion limit (SEL) of PD, usually in the form of ribonucleoprotein complexes (RNPs) [5,6,7].

CMV is one of the most widely spread plant viruses causing great economical losses infecting more than 1200 plant species including monocots and dicots with high economic importance (e.g., tomato, pepper, cucumber, spinach) [8,9,10]. The genome of CMV consists of three positive strand RNAs (named RNA1, RNA2 and RNA3) that encodes five viral proteins: 1a, 2a, 2b, MP and coat protein (CP). Plant virus genomes encode limited number of proteins to maintain all aspects of viral cycle, thus all five proteins of CMV have multiple functions. The 1a protein mainly serves as a helicase and forms the viral RNA replication complex with 2a protein. The 2b protein is the shortest and the most versatile: Its functions include suppressing RNA gene silencing and symptom induction. The main function of the CP is virus encapsidation, but also has a role in symptom formation, viral movement, aphid transmission and host range determination [11,12,13,14,15]. Previously, four out of five proteins of CMV were experimentally identified to undergo phosphorylation during infection. Phosphorylation of the N-terminal 126 amino acid of 2a protein is essential in forming the replicase complex of the virus [16]. In the case of 2b protein, even the specific residues were identified whose phosphorylation greatly affects the function and subcellular localization of the protein: Dual phosphorylated state at Ser40 and Ser42 residues inhibited the nuclear localization and siRNA binding activity of 2b [17,18]. The role of phosphorylation of CMV CP was recently demonstrated in symptom development and virion stability [19].

The structure of CMV MP has not been resolved yet, but specific functional domains were already determined. At the central region of the protein, a hydrophobic domain (between amino acids 86 and 118) was identified [20]. Supporting the idea that CMV MP facilitates virus spread by binding the viral genome, an RNA-binding domain (between amino acids 174–233), two putative zinc finger domains (between amino acids 126–146 and 157–194) and two nucleic acid-binding domains (between amino acids 134–138 and 164–168) were also identified [12,20,21,22]. These data suggest that CMV MP facilitates virus spread as an RNP complex. To allocate the viral genome from the infected cell to the neighboring cells, MP localizes to and increase the SEL of plasmodesmata [23]. Although direct interaction between MP and CP was not identified, several studies demonstrated an essential role of the CP in viral transport. Even if CP is required for cell-to-cell movement of the virus, by deleting the C-terminal 33 amino acids of the MP this requirement is abolished and results in a stronger RNA-binding affinity of the protein [24,25]. The requirement for compatibility between the C-terminal 29 aa of the MP and the C-terminal two-thirds of the CP for cell-to-cell movement was also demonstrated [26]. Moreover, amino acid 51 of the MP and amino acid 129 of the CP affect symptom development in bottle gourd [27]. Further studies confirm the role of MP in symptom development: Amino acids 51 and 240 were identified to influence subcellular distribution and chronic symptom induction on tobacco [28]. Amino acid 168 of MP and amino acid 267 of 2a protein were observed to interact indirectly and affecting the cell-to-cell movement and symptom dynamics on zucchini [29]. RNA-binding affinity of MP was affected when the interaction between the N-terminal 20 amino acids of MP and N-terminal 21 amino acids and the central GDD motif of 2a were altered. A highly conserved Ser at position 14 of MP had a critical role in this interaction, suggesting the role of phosphorylation in MP [30]. So far phosphorylation of CMV MP was demonstrated in transgenic tobacco plants, but its role in the virus cycle was not yet examined [31].

In the present study, we aimed to analyze the potential function of two feasible phosphorylation sites of the CMV MP. The Ser amino acids at 28 and 120 aa positions were replaced with alanine (A) and aspartic acid (D). Ser28 was essential for proper virus infection on *Nicotiana tabacum* cv. Xanthi, on cucumber and for the local lesion development on *Chenopodium murale*. The mutations of Ser28 abolished the proper plasmodesmata localization of the MP. 

## 2. Materials and Methods

### 2.1. Plasmid Constructions

The isolation of Rs-CMV, a subgroup I strain from *Raphanus sativus* and the descriptions of the infectious transcripts (pRs1, pRs2, pRs3) has been published previously [32]. Ala and Asp mutant RNA3 clones (MP/S28A, MP/S28D, MP/S120A, MP/S120D) were generated by PCR directed mutagenesis of pRs3. The forward and reverse primers used are detailed in Table S1. For the construction of MP/S28A, two segments of MP were amplified using MPSacIfor-MP/S28ADrev and MP/S28Afor-MPrev primer pairs, and for MP/S28D, MPSacIfor-MP/S28ADrev and MP/S28Dfor-MPrev primer pairs were used. For the construction of MP/S120A, two segments of MP were amplified by PCR using MPSacIfor-MP/S120ADrev and MP/S120Afor-MPrev primer pairs, and for MP/S120D, MPSacIfor-MP/S120ADrev and MP/S120Dfor-MPrev primer pairs were used. The full-length MP was amplified by overlap PCR using MPSacIfor-MPrev primer pair in all cases. The amplified segment contained the desired mutations, which were checked with digestion of BglII and SacI.

The green fluorescent protein (eGFP) fusion constructs used for intracellular localization experiments were created by overlap PCR technique, resulting in MP-eGFP, MP/S28A-eGFP and MP/S28D-eGFP. The nucleotide sequence of the primers used are detailed in Table S1. The identity of all the constructs were verified by nucleotide sequence determination.

### 2.2. Plant Inoculation and Symptom Detection

*Nicotiana benthamiana*, *Nicotiana tabacum* cv. Xanthi and *Chenopodium murale* plants were grown in environment-controlled growth chambers with long day conditions of 16 h light period at 23 °C and an 8 h dark period at 20 °C. *Cucumis sativus* ’Szenzáció’ plants were kept with the same lightning conditions, but at 26 and 23 °C, respectively.

*N. benthamiana* plants were mechanically inoculated with *in vitro* transcripts of the wild type (Rs-CMV) or the mutant RNA3s, namely MP/S28A, MP/S28D, MP/S120A, MP/S120D in the presence of the wild type RNA1 and RNA2 transcripts as described previously [32]. Viruses were purified three weeks after the inoculations and equal amounts (10 µg/mL) of the purified virions in sodium phosphate buffer (0.03 M Na_2_HPO_4_ pH: 8.7) were used for further inoculation experiments in the presence of carborundum [33]. *N. tabacum* cv. Xanthi and *Ch. murale* plants were inoculated at four-leaf stage, whereas in the case of *C. sativus,* the cotyledons were inoculated before the emergence of the young leaves. Plants were observed for symptom development for a five-week period. Mock inoculated plants were inoculated with buffer and carborundum.

### 2.3. Analysis of Plants

To verify the stability of the mutations, total RNA was extracted from the purified virions and from the upper non-inoculated leaves of *N. tabacum* cv. Xanthi and *C. sativus* with SV Total RNA Isolation Kit (Promega, Madison WI, USA) according to the manufacturer’s instructions. RT-PCR was carried out using primers amplifying the full-length ORF of CMV MP (Table S1) and One-Step RT-PCR Kit (Qiagen, Hilden, Germany). Prior DNA sequence determination the PCR products were purified with High Pure PCR Product Purification Kit (Roche, Basel, Switzerland). 

In case of *Ch. murale* the inoculated leaves were collected 3 days post inoculation (dpi). The data regarding the size of the necrotic lesions were processed using Image J software (version 1.52, NIH). Data were analyzed by Kolmogorov–Smirnov test, skewness and kurtosis for normality of the data, and Levene’s test for homogeneity of variances. To evaluate the differences between the areas of the induced local necrotic lesions one-way ANOVA model coupled with Games-Howell’s post hoc test were used. The statistical analysis was carried out using IBM SPSS Statistics 25 and MS Excel. Descriptive statistics and the statistical analysis are detailed in Table S2.

Virus accumulation in *C. sativus* cotyledons were assessed with tissue print immunoassay. Inoculated cotyledons were detached at 10 dpi and scraped with a sterile razor blade. The freshly cut surface was pressed onto a nitrocellulose membrane (GE Healthcare Bio-Sciences, Uppsala, Sweden) leaving a tissue print. The membrane was hybridized with anti-CMV CP antibody to detect virus spread. 

### 2.4. Protein Analysis, SDS-PAGE and Immunoblotting

Protein extracts from *N. tabacum* cv. Xanthi and *C. sativus* plants were prepared from 20 mg leaf samples. The leaf discs were grounded in 0.1 mL of 1x protein sample buffer in an ice-cold mortar. Prior to SDS-PAGE the samples were denatured at 95 °C for 5 min and centrifuged for 1 min. Five µl of the protein extracts were separated on 12% SDS-PAGE gels. The equivalence of the proteins was verified using Coomassie Brilliant Blue Staining G250. After electrophoresis proteins were transferred to a nitrocellulose membrane (GE Healthcare Bio-Sciences) and subjected to immunoblot analysis using CMV CP antibody. Goat anti-rabbit ALP conjugated IgG (Agrisera, Vännäs, Sweden) was used as secondary antibody and AP Conjugate Substrate Kit (Bio-Rad, Hercules CA, USA) was used for detection.

### 2.5. Agroinfiltration

For *Agrobacterium*-mediated transient expression assay MP, MP/S28A ad MP/S28D fused with eGFP were cloned into binary vector pBIN61s and transformed into *Agrobacterium* C58C3 strain. The *Agrobacterium* strain expressing p14 suppressor protein of cymbidium ringspot virus was described previously [34]. After growing overnight at 28 °C the bacterium cultures expressing wild type (MP-eGFP) and mutant MPs (MP/S28A-eGFP, MP/S28D-eGFP) were adjusted to a final OD_600_ to 0.4 and the *Agrobacterium* strain expressing p14 suppressor protein to 0.2. The agroinfiltration was carried out on *N. benthamiana* and *N. tabacum* cv. Xanthi leaves by pressure infiltration with a needleless syringe as described previously [31]. The infiltrated plants were at 6-leaf-stage and were kept in growth chambers (16 h light at 23 °C/8 h dark at 20 °C) for 24–48 h prior further analysis.

Callose at PD was stained with aniline blue (2:3 ratio of 0.1% aniline blue and 1 M glycine pH: 9.5) by infiltration into leaves 10 min before visualization [35]. For plasmolysis, leaf sections were incubated in 10% NaCl and the plasmolyzed cells were examined by confocal microscopy.

### 2.6. Confocal Microscopy 

Images were obtained using a Leica TCS SP8 confocal laser-scanning microscope (Leica Microsystems GmbH, Wetzlar, Germany). Images were acquired by a HC PL APO CS2 40×/1.10 water immersion objective. Confocal aperture size was set to 0.585 Airy Unit. Image acquisition was carried out by bidirectional scanning along the *x*-axis, and images were averaged from three distinct image frames in order to reduce image noise. Aniline blue dye was excited at 405 nm, staining pattern was detected between 410–480 nm. GFP was excited at 488 nm, the emitted fluorescence was detected between 490–530 nm.

## 3. Results

### 3.1. Mutations in the CMV MP

Rs-CMV MP was analyzed in silico to predict feasible phosphorylation sites (NetPhosK and ELM database) [36,37]. Ser residues in positions 28 and 120 were selected for further investigation.

In order to determine the significance of these potential phosphorylation sites in the virus life cycle, Ser in both aa positions (28 and 120) were substituted with alanine (A) modelling the non-phosphorylated state and with phosphorylation-mimicking aspartic acid (D). Mutations were introduced into Rs-CMV RNA3 infectious clones resulting the clones MP/S28A, MP/S28D, MP/S120A and MP/S120D. For inoculation of *Nicotiana benthamiana* plants in vitro transcripts of the wild type or the mutated RNA3 were combined with wild type RNA1, RNA2 in vitro transcripts. During the monitoring period (30 days) no significant difference was observed in the symptom development of the wild type and the mutant viruses. Virions were purified for further experiments and RT-PCR and nucleotide sequence determination confirmed the identity and stability of the purified mutant viruses even 30 days after the inoculation.

### 3.2. Systemic Symptom Development of MP/S28A, MP/S28D, MP/S120A and MP/S120D Mutant Viruses on Nicotiana tabacum cv. Xanthi

**Figure 1:**
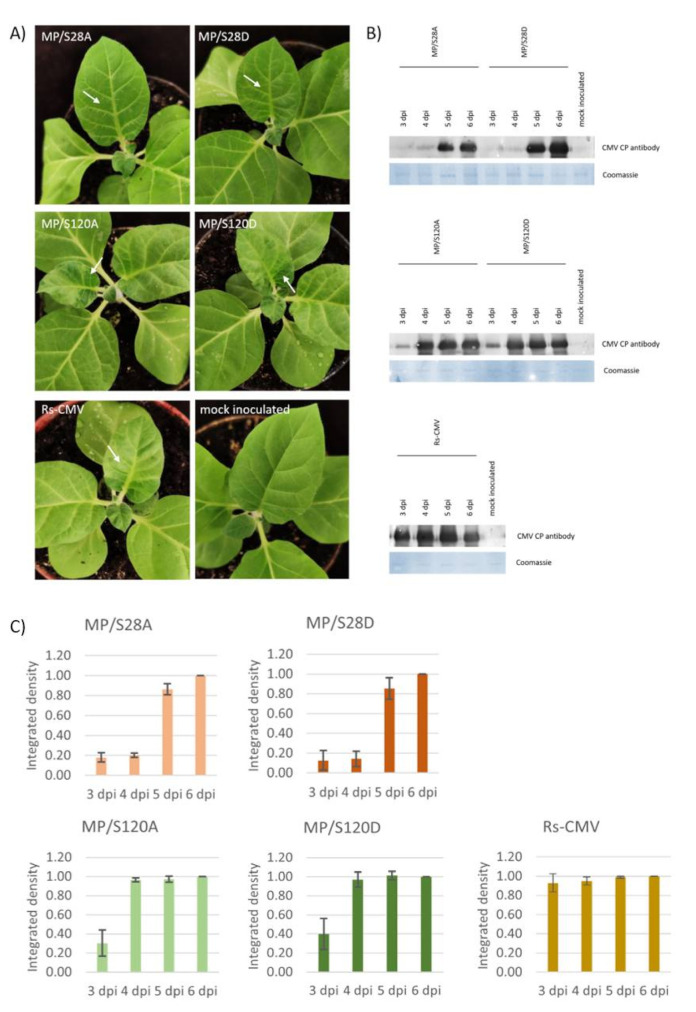
Symptom development on *Nicotiana tabacum* cv. Xanthi induced by MP/S28A, MP/S28D, MP/S120A, MP/S120D and wild type Rs-CMV. (**A**) Systemic symptoms on *N. tabacum* cv. Xanthi plants 5 days post inoculation (dpi). Arrows indicate the recognized symptoms. (**B**) Western blot analysis of coat protein (CP) accumulation in the systemically infected leaves 3–6 dpi. The equal protein loading was verified with Coomassie staining. (**C**) Bar diagrams representing the relative integrated density of the Western blot analysis. Standard deviations from three independent experiments are represented.

Systemic symptom development was monitored on the young non-inoculated leaves of *N. tabacum* cv. Xanthi plants. Wild type Rs-CMV induced systemic mosaic and slight distortion on the upper leaves of the tobacco plants 4 dpi and severe symptoms were recognized on the following days. MP mutants MP/S120A and MP/S120D elicited similar systemic symptoms although with one day shift compared to the Rs-CMV. In the case of MP/S28A and MP/S28D viruses significantly milder symptoms were developed. On 5 dpi (by the time Rs-CMV and 120 aa mutants both induced characteristic systemic symptoms) vein chlorosis was detected on the upper non-inoculated leaves (Figure 1A). The virus accumulation of wild type and mutant CMV was assessed with anti-CMV CP antibody in the 3-to-6-day period after inoculation. Three dpi high concentration of CP was detected in the upper leaves of the wild type CMV infected plant. In the case of MP/S120A and MP/S120D mutants, although the CP was detectable on 3 dpi, the concentrations were significantly lower compared to the Rs-CMV. On 4 dpi the virus titer was similar to the Rs-CMV. The CP of the mutant MP/S28A and MP/S28D viruses were first detectable 5 dpi in the upper non-inoculated leaves (Figure 1B,C). The milder symptom development and delayed systemic symptom emergence corresponded to the lower levels of virus accumulation detected by Western blot. RT/PCR and nucleotide sequence determination proved the identity of all the viruses used in this experiment 10 dpi.

### 3.3. Symptom Development on Cucumis sativus

**Figure 2:**
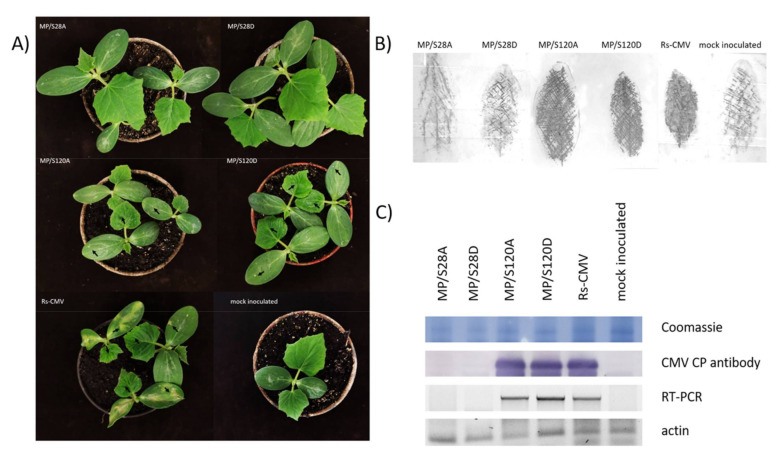
Symptom formation on *Cucumis sativus* plants 10 dpi inoculated with wild type Rs-CMV or movement protein (MP) mutants: MP/S28A, MP/S28D, MP/S120A, MP/S120D. (**A**) The cotyledons were inoculated with purified virions and systemic symptoms were observed. The mosaic symptoms on the young leaves and the local necrotic lesions on the cotyledons were marked with arrows. (**B**) Tissue print immunoassay of inoculated cotyledons at 10 dpi. (**C**) Systemic infection of the mutant viruses was analyzed by Western blot analysis and RT-PCR.

The symptom development and virus accumulation were also observed on an economically important host of CMV—*Cucumis sativus*. By 10 dpi the wild type CMV induced large necrotic lesions on the inoculated cotyledons and mosaic symptoms were also detected on the young non-inoculated leaves (Figure 2A). MP/S120A and MP/S120D virus infected plants elicited similar systemic symptoms (chlorotic mosaic and leaf distortions) with smaller necrotic lesions on the inoculated cotyledons. No systemic symptoms were observed on the cucumber plants inoculated with MP/S28A and MP/S28D viruses. To verify the visual observations, tissue print immunoassay of the inoculated cotyledons was carried out (Figure 2B). In the case of Rs-CMV, MP/S120A and MP/S120D the virus was evenly detected in the inoculated cotyledon, while mutants MP/S28A and MP/S28D were not detectable. For the upper, non-inoculated leaves RT-PCR and Western blot analysis were used for detection of virus accumulation (Figure 2C). In accordance with visual observations solely the Rs-CMV, MP/S120A and MP/S120D RNA and CP were detected in the upper leaves of *C. sativus*.

### 3.4. Local Lesion Induction on Chenopodium murale

**Figure 3:**
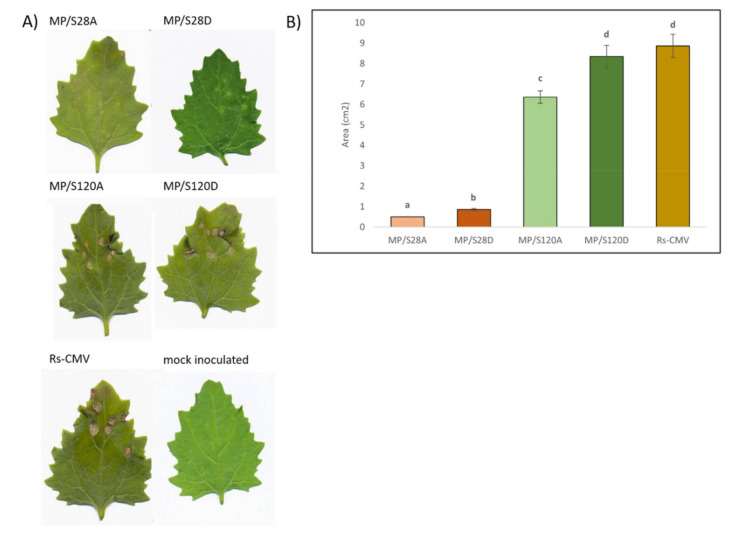
Local lesion development on *Chenopodium murale* 4 dpi with Rs-CMV and the MP mutants. (**A**) Local lesions on the inoculated leaves of Rs-CMV, MP/S28A, MP/S28D, MP/S120A and MP/S120D. (**B**) Diagram demonstrating the statistical analysis of lesion sizes (with standard deviation) induced by the different MP mutants and the wild type Rs-CMV.

*Chenopodium murale* is a local lesion host for CMV and so hypersensitive response is induced on the inoculated leaves. In the present experiment, the local symptoms were quantified at 4 dpi (Figure 3A). Wild type Rs-CMV induced large necrotic local lesions on the inoculated leaves of *Ch. murale*. MP/S28A and MP/S28D caused much smaller lesions and inoculation with MP/S120A and MP/S120D viruses resulted in similar lesion sizes and phenotype as wild type CMV. The difference between the induced local symptoms was quantified based on the areas of the local necrotic lesion on the inoculated leaves (Figure 3B). Significant differences were found among the values for the individual viruses (*P* < 0.0001). MP/S120D induced necrotic lesions with no significant difference in size to wild type CMV. MP/S120A caused visually barely observable but statistically demonstrable difference in lesion size compared to wild type CMV. The necrotic lesion sizes induced by mutants MP/S28A and MP/S28D were significantly smaller than the lesions of any other mutants or wild type CMV.

### 3.5. PD Localization is Affected by Mutations of Ser28 of CMV MP

**Figure 4:**
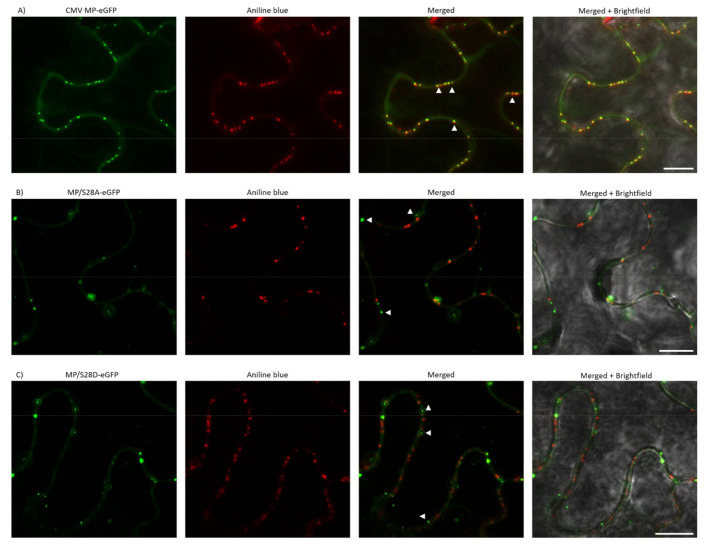
Subcellular localization of wild type and mutant MP-eGFP constructs on *Nicotiana benthamiana*. (**A**–**C**) MP-eGFP and mutant constructs MP/S28A-eGFP and MP/S28D-eGFP were transiently expressed in *N. benthamiana* leaves. Aniline blue staining served as a plasmodesmata marker. Images were taken 24–48 h after agroinfiltration using a confocal microscope. For better visualization, eGFP fluorescent and aniline blue images were false colored as green and red, respectively. Arrowheads indicate the punctate structures. Scale bar: 10 µm.

**Figure 5:**
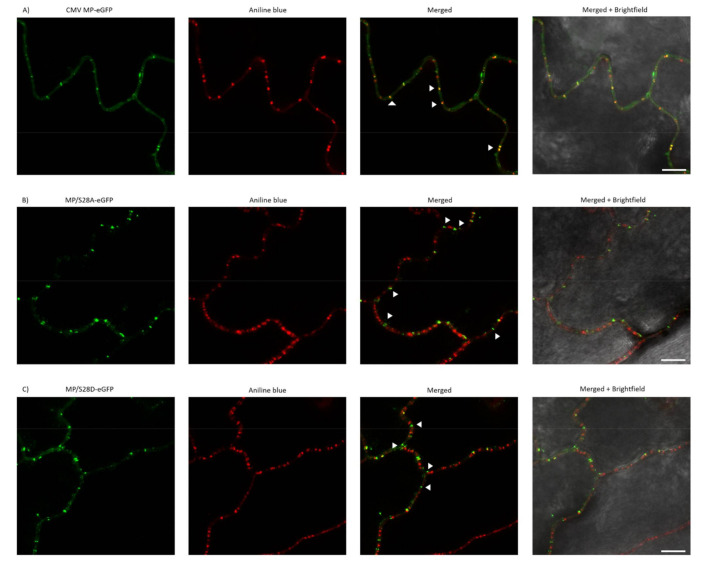
Subcellular localization of wild type and mutant MP-eGFP constructs in *Nicotiana tabacum* cv. Xanthi leaves. (**A**–**C**) Aniline blue staining served as a plasmodesmata marker. Images were taken 24–48 h after agroinfiltration with a confocal microscope. For better visualization, eGFP fluorescent and aniline blue images were false colored as green and red, respectively. Arrowheads indicate the punctate structures. Scale bar: 10 µm.

**Figure 6:**
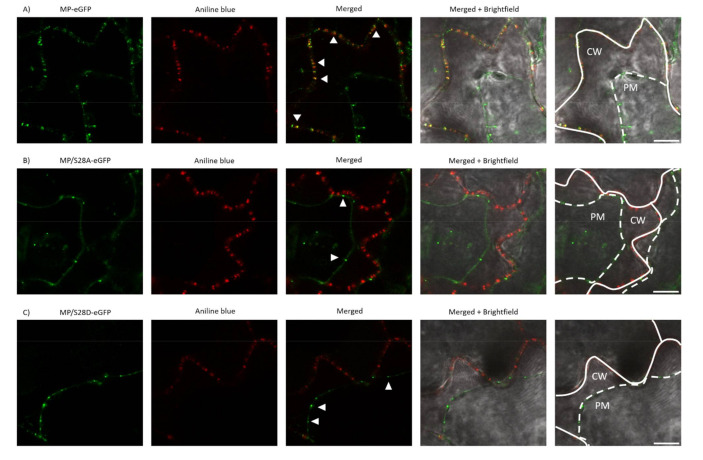
Subcellular localization of wild type and mutant MP-eGFP constructs in aniline blue stained *Nicotiana tabacum* cv. Xanthi leaves. (**A**–**C**) Images were taken 24–48 h after agroinfiltration with a confocal microscope. For plasmolysis treatment, leaves were soaked in 10% NaCl solution immediately before examination. For better visualization, eGFP fluorescent and aniline blue images were false colored as green and red, respectively. The plasma membrane (PM) and the cell wall (CW) were marked by white dashed and continuous lines, respectively. Arrowheads indicate the punctate structures. Scale bar: 10 µm.

As MP/S28A and MP/S28D viruses induced highly distinctive local and systemic symptoms compared to wild type CMV, the subcellular localization of 28 aa mutant viruses was examined. In previous studies, CMV MP fused with a fluorescent protein to its C-terminus was found to be functional [38] and showed subcellular localization at PD. In the present study, eGFP was fused to the C-terminus of wild type MP and the mutated MP constructs as well, resulting in MP-eGFP, MP/S28A-eGFP and MP/S28D-eGFP. *Nicotiana benthamiana* and *Nicotiana tabacum* cv. Xanthi epidermal cells were assayed 24–48 h after agroinfiltration with a confocal laser-scanning microscope. In the case of MP-eGFP punctate-like distribution pattern along the cell wall was detected on both plants. After staining the leaf samples with aniline blue, the two fluorescent signals greatly coincided, thus confirming PD localization of the MP. However, when Ser28 residue was mutated to Ala or Asp the localization pattern was clearly distinct. Unlike wild type MP-eGFP, MP/S28A-eGFP and MP/S28D-eGFP barely co-localized with aniline blue and the punctate spot localization pattern of the mutant constructs was markedly different as the PD distribution in the examined cells (Figure 4 and Figure 5). To further verify the localization patterns, we used plasmolysis, which separates cell-wall associated structures (for example plasmodesmata) from other intracellular compartments. The green fluorescent signal of MP-eGFP were co-localized to the punctate structures of aniline blue stained callose, indicating strong PD localization along the cell wall (Figure 6A). However, the fluorescent signals of both MP/S28A-eGFP and MP/S28D-eGFP retracted with the displaced cellular structures inside the constricted plasma membrane and formed only occasional plasmodesmal distribution (Figure 6B,C). These experiments confirmed that although MP/S28A and MP/S28D mutations do not abolish completely, they do highly affect the efficient PD localization of the MP, and in consequence, the cell-to-cell movement of the virus could be affected.

## 4. Discussion

Post-translational modifications (PTMs) are reversible modifications playing a fundamental role in protein function regulations. PTMs can alter protein function, stability, localization, and protein–protein interactions, which provides functional diversification of protein activity in eukaryotic cells [39]. Most common types of PTMs are phosphorylation, alkylation, glycosylation, acylation, oxidation, and methylation. According to statistical analysis based on Swiss-Prot database, phosphorylation is the number one PTM in frequency considering both the experimentally described and putative PTM sites [40].

The only data available on the phosphorylation of CMV MP are from Matsushita et al. [31]. In transgenic MP-expressing tobacco lines, [32P]-orthophosphate was identified to associate with the MP, but the position of the phosphorylation was not determined. Moreover, this study revealed that only phosphoserine, not phosphothreonine or phosphotyrosine, was present in CMV MP. It was the first confirmation of CMV MP phosphorylation *in planta*, but the possible role of phosphorylation in the CMV infection cycle was not analyzed. In silico evaluation of possible phosphorylation sites of CMV MP revealed that Ser residue at positions 28 and 120 might be phosphorylatable. Phylogenetic analysis indicated that Ser28 is highly conserved in the genus *Cucumovirus* (Figure S1). It is present in the MP of both subgroups of CMV (subgroups I and II) and in the MP of other members of the genus, tomato aspermy virus (TAV) and peanut stunt virus (PSV) as well, supporting the functional relevance of Ser28. This Ser28 does not overlap with any of the previously identified functional domains of the MP. The domain responsible for the interaction with 2a protein (1-20aa) has the closest location, but no other general functional domain in the N-terminal region preceding the central hydrophobic core domain was identified. Interestingly, the phosphorylation status of Ser14 in the 2a protein-interacting domain of CMV MP was speculated to determine the interaction [30]. This Ser14 is also highly conserved among cucumoviruses. The viral RNA genome plasticity is a key factor of evolutionary success of RNA viruses, but in some cases remarkable stability can be observed, since the conservation of certain domains responsible for specific functions have a key role in viral life cycle.

In this study, the functional impact of two potential phosphorylatable Ser residues of the CMV MP were analyzed by mutagenesis; Ser at positions 28 and 120 were substituted with Ala or Asp (mimicking the non-phosphorylated or phosphorylated states, respectively), and significant difference between systemic symptoms was observed. In symptom development of the MP/S120A and MP/S120D mutants, just slight modification in symptom timing and formation was observed. The belated symptom development on *Nicotiana tabacum* cv. Xanthi, the minor (but demonstrable) difference in the elicited necrotic lesions on *Chenopodium murale* and the difference in the local lesion phenotype on the inoculated cotyledon of *Cucumis sativus* indicate a minor effect of the mutations in Ser120 aa. Although the present study focused on the more apparent effects of Ser28 on symptom formation, further study could be conducted in the future to explore the explicit role of Ser120 and even the complex phosphorylation pattern could be analyzed. Significant impact was recognized in the case of the MP/S28A and MP/S28D on hosts tested: On *N. tabacum* cv. Xanthi the delay of systemic symptom development was observed, on *Ch. murale* the induced lesion sizes were substantially smaller compared to wild type CMV and on *C. sativus* no virus accumulation was detected neither in the inoculated cotyledons nor in the upper non-inoculated leaves. Previously, the effect of mutations in the phosphorylation site of MP on virulence was suggested to be host specific [41], but in the present study, Ser28 mutation has general effect on the three different hosts tested.

The effect of MP phosphorylation on pathological properties of plant viruses was recognized just in a few cases. *In vivo* and *in vitro* phosphorylation of tobacco mosaic virus (TMV) MP was identified at positions Ser258, Thr260 and Ser265 residues. Although deleting the C-terminal 55 amino acids of TMV MP (including these three putative phosphorylation sites) did not abolish virus spread in tobacco (both *N. tabacum* and *N. benthamiana*) [42,43,44], aspartic acid mutants of these three residues showed host-dependent negative effect on viral spread in *N. tabacum*, but not in *N. benthamiana* [45,46]. In the case of tomato mosaic virus (ToMV) MP, two phosphorylation sites were identified (Ser37, Ser238) to play an important role in symptom induction. In the C-terminal region, Ala substitution of Ser238 had a minor effect, since it only induced smaller local necrotic lesions on inoculated leaves of *N. tabacum* cv. Xanthi [47]. On the other hand, in the amino-terminal region of ToMV, when substituting Ser37 to Ala, necrotic lesion induction was completely abolished on *N. tabacum* cv. Xanthi and no symptoms were observed on systemic hosts, like *N. tabacum* cv. Samsun and tomato plants [47]. In the case of potato mop top virus (PMTV), Ala mutants of Tyr87-89 of TGBp3 inhibited systemic spread in *N. benthamiana* host and displayed a negative effect on protein interaction with TGBp2 [48]. In the case of abutilon mosaic virus (AbMV), connection was demonstrated between phosphorylation of MP (at Thr221, Ser223 and Ser250 residues) and symptom induction and virus titer in *N. benthamiana* [49]. In the case of barley stripe mosaic virus (BSMV), two putative threonine phosphorylation sites were identified altering symptom induction on different hosts. Infectivity experiments proved that the non-phosphorylated state only hampered systemic infection in the case of Thr401, and only in dicot (*N. benthamiana*) and not in monocot hosts (barley, wheat). Meanwhile Asp mutation of both Thr395 and Thr401 abolished systemic infection in monocot and dicot hosts as well [50]. Our results are in agreement with these findings and expand the number of viruses where point mutations in possible phosphorylation sites of viral MPs can induce host specific differences of symptom developments.

The main function of viral MPs is the facilitation of cell-to-cell movement of the virus. Among the members of ‘30K superfamily’, MPs complete this function by localizing to PD and increasing its SEL. Phosphorylation of the C-terminal region of MP was demonstrated to influence the subcellular localization of tobamoviruses TMV and ToMV. As to TMV, three putative C-terminal phosphorylation sites (Ser288, Thr261, Ser265) were substituted to phosphorylation-mimicking Asp residue, which reduced its ability to localize to PD (thus facilitating viral spread) in *N. tabacum* [51]. ToMV MP S238A mutant showed less distinct plasmodesmata localization pattern compared to wild type ToMV MP in tobacco [47]. Phosphorylation in the amino-terminal region of the MP was also identified as important factor of plasmodesmata localization. ToMV MP S37A mutant was unable to localize to plasmodesmata and showed a dispersed cytoplasmic localization pattern [47]. In case of potato leaf roll virus (PLRV), PD targeting of MP is regulated by possible sequential phosphorylation of Ser71 and Ser79 residues [52].

In our experiments, in accordance with previous results, the wild type CMV MP-eGFP localized in a plasmodesmata-specific punctate pattern. The subcellular localizations of the amino-terminal mutants MP/S28A-eGFP and MP/S28D-eGFP were distinct, they were not localized with callose at PD, but in punctate spots along the cell periphery. Plasmolysis experiments demonstrated that 28 aa mutants mainly associated with the displaced membrane rather than the cell wall, indicating that phosphorylation of CMV MP at Ser28 position affects PD localization. The fact that both the Ala and Asp mutants failed to show a PD localization pattern suggests that phosphorylation at this residue does not have an on-and-off switch-like effect on protein function, rather the dynamic interchange between the phosphorylated and non-phosphorylated state may play a crucial role in adequate subcellular localization. The determination of the kinase targeting the Ser28 of CMV MP requires further work in the future, and the subcellular localization of the kinase could facilitate the understanding of the complete mechanism of MP localization. The kinase responsible for MP phosphorylation was determined in a few cases. The MPs of TMV and bean dwarf mosaic virus (BDMV) are phosphorylated by a protein kinase belonging to the casein kinase 1 family, the plasmodesma-associated protein kinase (PAPK), which is observed to be corresponding with these two MP’s intracellular localization signals [53].

Our results indicate a new important factor in plasmodesmata localization of CMV MP, which have an effect in symptom development in several hosts plants. The fact that Ser28 is generally present among cucumoviruses and its mutants displayed demonstrable differences in symptom development dynamics of both systemic and necrotic hosts of CMV, reveals the importance of a new previously unknown putative phosphorylation site of CMV MP, which affects the plasmodesmata localization of the protein. The understanding of the complex process of plasmodesmata targeting of viral MPs requires further study, so it is of primary interest to analyze the details of the interactions between MPs and plant host proteins during PD localization. The first plant viral plasmodesmata localization signal (PLS) was identified recently in TMV. The first N-terminal 50 aa was necessary and sufficient for PD targeting. [54]. Furthermore, the interaction of TMV PLS with plant synaptotagmin (SYTA) was also demonstrated [55]. In the future, investigations of the details of viral MP and PD interactions will enhance our knowledge on general virus-plant interactions.

## Data Availability

Data sharing is not applicable to this article.

## Supplementary Materials

The following are available online at https://www.mdpi.com/1999-4915/13/2/222/s1, Table S1: Primer sequences for cloning. The codons containing the mutations are underlined while the restriction enzymes used are in italics. Table S2: Descriptive statistics, Levene’s Test of Equality of Error Variances and Tests of Normality of the dataset examined.
