# Supplementary material for: The 28 Ser Amino Acid of Cucumber Mosaic Virus Movement Protein Has a Role in Symptom Formation and Plasmodesmata Localization
Source: Viruses. 2021 Jan 31;13(2):222. doi: 10.3390/v13020222 (PMC7912182; doi:10.3390/v13020222)
Supplement: Supplementary file 1 [file viruses-13-00222-s001.pdf]

Table S1. Primer sequences for cloning. The codons containing the mutations are underlined while the restriction enzymes used are in italics.

Primer	Sequence (5’-3’)
MP/S28Afor	GG*AGATCT*TATTTGCCCCTGAAGCCATTAAGAAAATGGC
MP/S28ADrev	GG*AGATCT*TTTGAAGATCGTCAGACGTATCCGCTGAGG
MP/S28Dfor	GG*AGATCT*TATTTGACCCTGAAGCCATTAAGAAAATGGC
MP/S120Afor	GG*GAGCTC*GCTCCCATAGATGGGCAATGCGTTTCG
MP/S120ADrev	GG*GAGCTC*CTTGTCGCCTAGATCAGCTAAGTAAATTCTCAA
MP/S120Dfor	GG*GAGCTC*GATCCCATAGATGGGCAATGCGTTTCG
MPrev	GCC*GGATCC*CTAAAGACCGTTAACCACCTGC
MPSacIfor	GG*GAGCTC*ATGGCTTTCCAAGGTACCAGT
MPeGFPrev	CGCCCTTGCTCACCATAAGACCGTTAACCACCTGC
MPeGFPfor	GTGGTTAACGGTCTTATGGTGAGCAAGGGCGAGG
eGFPBamHIrev	CC*GGATCC*TCACTTGTACAGCTCGTCCATG
CMVMPpETfor	GG*CATATG*GCTTTCCAAGGTACCAGTAG
CMVMPpETrev	GCC*GGATCC*CTAAAGACCGTTAACCACCTGC
Cap. actin for	AGGGATGGGTCAAAAGGATGC
Cap. actin rev	GAGACAACACCGCCTGAATAGC

Table S2. Descriptive statistics, Levene's Test of Equality of Error Variances and Tests of Normality of the dataset examined

**Descriptive Statistics**			
Mutants	Mean	Std. Deviation	N
MP/S28A	0.499	0.015	15
MP/S28D	0.861	0.052	15
MP/S120A	6.363	0.304	15
MP/S120D	8.344	0.552	15
Rs-CMV	8.869	0.571	15
Total	4.987	3.660	75
**Levene's Test of Equality of Error Variances**			
F	df1	df2	Sig.
1688.542	4	70	0.000
**Tests of Normality**			**Skewness**
Kolmogorov-Smirnov			0.550
Statistic	df	Sig.	**Kurtosis**
0.203	100	0.000	0.926

Figure S1. Sequence comparison of the 1-50 amino acids of plant viruses in the genus Cucumovirus. Ser 28 is indicated with a red rectangle. PSV: Peanut stunt virus, TAV: Tomato aspermy virus.

CMV-Rs (sg I)	MAFQGTSRTLTQQSSAATSDDLQKILTSPEAIKKMATECDLGRHHWMRAD
CMV-Tsh(sg II)	MAFQGTSRTLTQQSSAASSDDLQKILTSPDAIKKMATECDLGRHHWMRAD
PSV	MAFSGSSRTLTQQSSAATSDDLHRILTSPEAIKEMATKCDLGRHHWMRAD
TAV	MAFSGTSRTLSQQSSAASTDELHNILTSRRAIQEMATKCDLGRHHWMRAD
